# Utility of Gadolinium Use in the Imaging Follow-Up of Nonenhancing Primary Brain Neoplasms in Children

**DOI:** 10.7759/cureus.31531

**Published:** 2022-11-15

**Authors:** Tiagpaul Bhamber, Zereen Sarwar, Yekaterina Jones, Brittany K Albers, Chetan Shah

**Affiliations:** 1 Radiology, University of Florida College of Medicine, Gainesville, USA; 2 Radiology, University of Florida College of Medicine-Jacksonville, Jacksonville, USA; 3 Pediatric Radiology, Norton Children's Hospital, Louisville, USA; 4 Pediatric Radiology, Nemours Children's Health System, Jacksonville, USA

**Keywords:** pediatric brain mri, gadolinium retention, mri protocol, mri contrast, nonenhancing brain neoplasm, mri, gadolinium

## Abstract

Background and purpose

Gadolinium-based contrast agents (GBCAs) have been administered clinically since 1988. They are remarkably well tolerated by children and result in dose-dependent tissue deposition, even in patients with normal renal function. No adverse effects of gadolinium deposition in patients with normal renal function have been established. Given the uncertain effects of gadolinium deposition, we sought to analyze gadolinium use in the imaging follow-up of nonenhancing primary brain neoplasms in children.

Materials and methods

This retrospective, institutional review board-approved and Health Insurance Portability and Accountability Act-compliant study evaluated pediatric patients who received GBCA in the routine evaluation of brain neoplasms. This special subset included 30 patients (<18 years old) with initially nonenhancing primary intracranial neoplasms who received treatment and follow-up at our institution. Patient data included sex, age from diagnosis to most recent imaging follow-up, number of contrast-enhanced magnetic resonance imaging (MRI) follow-up exams, and histopathology from a biopsy or resection.

Results

The group had an expected variety of tumors, including low-grade astrocytomas, dysembryoplastic neuroepithelial tumors, oligodendrogliomas, and teratomas. Half of our patients had tumors of unknown histopathology that were not biopsied or resected. The median age at diagnosis was 8.9 years, the median of four follow-up MRIs per patient, and the median follow-up time of four years. Only one of the 30 patients developed an enhancing focus on follow-up MRI that remained stable and asymptomatic over two years and did not require surgical intervention.

Conclusion

Judicious use of GBCA in children, especially when numerous exams over many years are anticipated, is advised given the data regarding soft-tissue deposition. Preliminary results suggest that it may be feasible to omit GBCA from routine follow-ups of initially nonenhancing brain neoplasms.

## Introduction

Incidence of pediatric brain tumors ranges between one and eight cases per 100,000 children. Posterior fossa tumors are more common in children. Medulloblastoma, ependymoma, and juvenile pilocytic astrocytoma are the three most common posterior fossa tumors in children. Gadolinium-based contrast agents (GBCAs) have been a mainstay of pediatric magnetic resonance imaging (MRI) for years and have been used in neuroimaging since the 1980s [1,2]. Like the adult population, patients are screened for renal function prior to receiving GBCA because of the risk of developing nephrogenic systemic fibrosis (NSF) [1-3]. Luckily, the incidence of pediatric NSF is incredibly low. Otherwise, GBCAs are well tolerated by children, with a low rate of allergic reaction and no appreciable short-term effect on renal or liver function [4,5]. Recent literature has established that the administration of GBCA results in gadolinium deposition in the brain, particularly in the globus pallidus and dentate nucleus [6-11], even in patients with normal renal function. While gadolinium deposition has been shown, no adverse effects in patients with normal renal function have been documented in any patient population [2,7-9,11-14]. We sought to analyze our GBCA use to see what clinical benefit the post-contrast imaging provided. The target patient population was a group of patients who received numerous follow-up exams, often over several years. We chose children with central nervous system (CNS) neoplasms.

## Materials and methods

This retrospective, institutional review board-approved and Health Insurance Portability and Accountability Act-compliant study evaluated pediatric patients who received GBCA in the routine evaluation of CNS neoplasms. An interesting group of patients who had primary CNS tumors that did not initially enhance was segmented and evaluated.

The images were reviewed by a pediatric fellowship-trained board-certified radiologist with certification in pediatric radiology. Images were reviewed unbiasedly without access to radiology reports or medical records. Findings were entered into a spreadsheet. Data were compared by the radiology resident with the patient's medical record and radiology report. Pathology reports were reviewed to gather information on the histopathological diagnosis.

We sought to determine what, if any, benefit was garnered by administering GBCA for follow-up MRI in patients whose tumors did not initially enhance. Inclusion criteria included the patient age being less than 18 years old, having a primary intracranial neoplasm that did not enhance, and having received follow-up at our institution. All the MRIs were performed with and without the administration of gadolinium-based contrast agents. The MRI protocol included 3D T2-weighted, fluid-attenuated inversion recovery (FLAIR), diffusion-weighted, and pre- and post-contrast 3D T1-weighted sequences. Perfusion images were not obtained. Thirty patients were included.

All the MRIs were evaluated with a focus on any incremental information that could not be seen on the noncontrast portion of the MRI. Any incremental information on contrast images was evaluated for its clinical significance and any effect on patient care.

## Results

**Figure 1:**
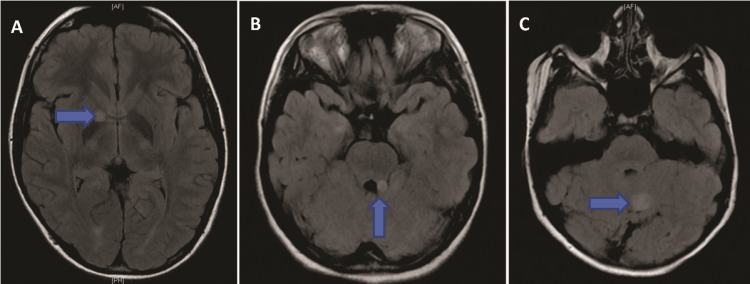
Axial fluid-attenuated inversion recovery images. Axial fluid-attenuated inversion recovery images from three different patients highlight the typical appearance of tumors of unknown histopathology (arrows) as seen in the right globus pallidus (A), the left middle cerebellar peduncle (B), and the cerebellar vermis (C). There is a flattening of the left lateral wall of the fourth ventricle by lesion (B), showing a focal mass effect.

There were 11 girls and 19 boys in the cohort, including newborns and those under 18 years of age, with a median age of 8.9 years. These patients received anywhere from one to 21 follow-up exams, with a median of four contrast-enhanced MRIs per patient. The median follow-up time was four years. The group had an expected variety of tumors, including low-grade astrocytomas, dysembryoplastic neuroepithelial tumors, oligodendrogliomas, and teratomas (Table 1). Half of our patients had “tumors of unknown histopathology.” These tumors included those for which a biopsy or excision was not performed. As the lesions were asymptomatic and these findings were incidental, a biopsy was deemed unnecessary, and hence close observation was done using follow-up MRIs. Heterotopias usually have similar signal intensity as gray matter, whereas these lesions were hyperintense compared to gray matter on the FLAIR sequence. Thus, these lesions were felt less likely to be heterotopias. All these tumors can be described as having a mass effect and altered signal intensity (Figure 1), which is concerning for low-grade gliomas. None of the patients had extracranial malignancies. None of these tumors resolved or regressed on follow-up imaging. These patients are under clinical and imaging surveillance.

**Table 1:** Distribution of neoplasms.

Type of neoplasm	Number of cases
Brainstem glioma	4
Low-grade astrocytoma	3
Dysembryoplastic neuroepithelial tumor	3
Oligodendroglioma	3
Anaplastic astrocytoma	1
Teratoma	1
Tumor of unknown histology	15

**Figure 2:**
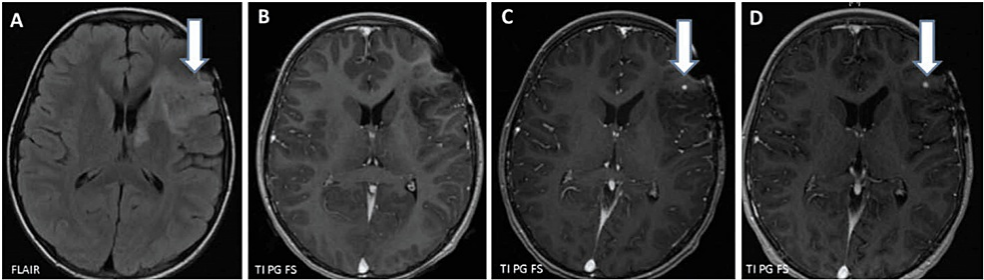
Axial MRI images. Initial axial MRI (A) shows a hyperintense left frontal lobe oligodendroglioma that was resected at age six years. No post-contrast enhancement was seen in the resection bed on the T1-weighted post-gadolinium axial image two years after the surgery. T1-axial post-contrast axial MRI image (C) performed six months after image (B) shows a tiny 2-mm enhancing focus (arrow) in the surgical bed that remained stable even after two more years (D). MRI: magnetic resonance imaging.

One patient had contrast enhancement that was eventually detected on follow-up (Figure 2). This patient had a left frontal oligodendroglioma resected at age six years. Three years after resection, expected gliosis about the operative cavity was seen on an axial fluid-attenuated inversion recovery image. No contrast enhancement was present at that time. Six months later, during a routine follow-up, a 2-mm nodule of enhancement was detected along the resection cavity. The 2-mm-enhancing nodule is stable nearly two years later, and the patient remains asymptomatic. None of the remaining 29 patients developed pathologic enhancement to suggest a residual or recurrent tumor during the follow-up time.

## Discussion

Gadolinium-based contrast agents were introduced over 30 years ago. Since then, there have been some concerns regarding their safety profile. In 2006, GBCAs were first linked to the development of NSF in patients on hemodialysis for renal impairment. These patients were found to have higher mortality [1,2]. To date, there is no established evidence that links GBCA to NSF in patients with normal renal function [2,7-9,11-14]. However, recent studies have found gadolinium deposition in various tissues. In 2009, gadolinium was found in bone tissue from the femoral heads of patients, even eight years after receiving GBCA prior to hip replacement surgery. This deposition did not have clinical significance, but it raised concerns about the long-term accumulation of gadolinium in patients who undergo multiple contrast-enhanced MRIs [3].

In 2010, insoluble deposits of gadolinium were discovered in biopsies from human brain tumors in 28 adult patients with no evidence of renal disease. Higher concentrations of gadolinium were observed in more vascularized areas of the biopsies, particularly within blood vessel walls [6]. In 2014, a postmortem study compared brain tissue samples from 13 deceased adult patients who had received between four and 29 gadolinium-enhanced MRIs with samples from 10 deceased patients who had never received GBCA. None of the patients involved in the study had any evidence of hepatic or renal impairment. The results showed a dose-dependent relationship between the number of contrast-enhanced MRIs each patient received and the amount of gadolinium deposition found in their brain tissue. This deposition was concentrated primarily in the globus pallidus and dentate nucleus [7]. In 2020, researchers conducting a postmortem analysis of 10 pediatric patients between 1 year and 13 years of age who had received at least one contrast-enhanced MRI found gadolinium deposition in tissue from all patients, particularly in the globus pallidus [8].

To our knowledge, there has been no study that examines the enhancement of primary nonenhancing CNS tumors in pediatric patients who receive multiple gadolinium-enhanced MRIs on follow-up visits. Our findings reveal that all but one patient did not develop pathologic enhancement suggestive of residual or recurrent tumor. One patient who developed a focus of enhancement within the previously nonenhancing lesion was observed over two years. The size of the lesion did not change even when the lesion was enhanced. The lesion remained stable over two years and did not require any surgical intervention. It is unlikely that this is a post-surgical change, as there was no enhancement in that region for two years after surgery. Hence, this new enhancement was presumed to be neoplastic and observed so as to err on the side of caution. This suggests that GBCA may not be necessary for follow-up imaging in these patients, thereby allowing them to avoid any potentially harmful effects of GBCA. A larger percentage (50%) of these lesions are tumors of unknown histopathology, which are usually observed and remain stable over time. We routinely encounter these lesions, which are usually not biopsied or resected and require multiple follow-ups to monitor for growth. None of these patients in our study showed enhancement on follow-up, and hence we suggest omitting GBCA in the follow-up of these lesions. There are additional benefits of excluding post-contrast imaging, including decreased exam time and cost. Also, there would not be a need for painful intravenous access in nonsedated children providing better patient compliance.

Clinicians should weigh the risks and benefits of GBCA in young patients who receive multiple follow-ups MRIs. Although there has yet to be evidence suggestive of clinical symptoms resulting from gadolinium deposition in the brain, there is evidence for a dose-dependent relationship between GBCA exposure and gadolinium deposition in essential brain structures, such as the globus pallidus and dentate nuclei [6-11]. Children have a potentially longer life span remaining and potentially receive more gadolinium over their lifetime. Currently, we have no knowledge of the effects of retained gadolinium in the body for 50 years or more, as gadolinium injections started about 34 years ago, in 1988. Additionally, it is well established that pediatric patients are more likely to experience the neurotoxic effects of other heavy metals when compared with adults. It is possible that this susceptibility could extend to GBCA. Therefore, clinicians should strive for the judicious use of GBCA. Future long-term and larger-scale research are needed to truly elucidate the potentially toxic effects of repeated GBCA exposure.

A limitation of the study is the small sample size. Future studies may be needed with a larger sample size and a multi-site study. Future research needs to evaluate the extent of gadolinium deposition in children. Additionally, the effect of treatment with chemotherapy and radiation on the accumulation of gadolinium should be evaluated. Controlled studies with careful standardization and documentation of GBCA formulation and dose, as well as imaging parameters such as scan time delay, would be helpful to confirm our findings.

## Conclusions

Since the long-term effect, if any, of gadolinium deposition has not been established, it is perhaps advisable to use GBCA judiciously. Given the dose-dependent nature of the accumulation of gadolinium, the limitation of gadolinium in patients expected to receive long-term follow-up may be appropriate. Our limited study indicates that patients with a nonenhancing primary intracranial neoplasm may be able to have their routine imaging follow-up without contrast, especially for nonenhancing neoplasms that are usually not biopsied or resected.
